# The Membrane Associated RING-CH Proteins: A Family of E3 Ligases with Diverse Roles through the Cell

**DOI:** 10.1155/2014/637295

**Published:** 2014-10-29

**Authors:** Tasleem Samji, Soonwook Hong, Robert E. Means

**Affiliations:** ^1^Department of Immunology, University of Connecticut Health Center, L3056, Mc1319, 263 Farmington Avenue, Farmington, CT 06030, USA; ^2^Department of Cellular and Molecular Physiology, Yale School of Medicine, 333 Cedar Street, New Haven, CT 06520, USA; ^3^Department of Pathology, Yale School of Medicine, LH315B, 310 Cedar Street, New Haven, CT 06524, USA

## Abstract

Since the discovery that conjugation of ubiquitin to proteins can drive proteolytic degradation, ubiquitination has been shown to perform a diverse range of functions in the cell. It plays an important role in endocytosis, signal transduction, trafficking of vesicles inside the cell, and even DNA repair. The process of ubiquitination-mediated control has turned out to be remarkably complex, involving a diverse array of proteins and many levels of control. This review focuses on a family of structurally related E3 ligases termed the membrane-associated RING-CH (MARCH) ubiquitin ligases, which were originally discovered as structural homologs to the virals E3s, K3, and K5 from Kaposi's sarcoma-associated herpesvirus (KSHV). These proteins contain a catalytic RING-CH finger and are typically membrane-bound, with some having up to 14 putative transmembrane domains. Despite several lines of evidence showing that the MARCH proteins play a complex and essential role in several cellular processes, this family remains understudied.

## 1. Introduction

Ubiquitination is a highly important mechanism for posttranslational control of protein functionality. Since its discovery, it has been shown to be involved in a wide variety of processes besides its originally identified role in protein degradation via the ubiquitin-proteasomal system, including endocytosis, signal transduction, protein translocation across membranes, intracellular protein transport, and even DNA repair.

Protein ubiquitination requires a cascade of three steps; first, the ubiquitin must be “activated” by an E1 ubiquitin-activating enzyme. The E1 hydrolyzes ATP to adenylate the ubiquitin C-terminal carboxyl group, forming an intermediate with a high-energy mixed anhydride bond that is quickly attacked by a cysteine on the E1. This creates a thioester bond between the E1 and the ubiquitin and also releases AMP. The activated ubiquitin is then transferred to the catalytic cysteine group of an E2 ubiquitin-conjugating enzyme via a trans-thiolation reaction. In the final step for the RING-CH class of E3 ligases, the ubiquitin is transferred to its substrate at the site of a lysine, cysteine, serine, or threonine residue with the aid of the E3 enzyme. Ubiquitin can be attached at a single site on a protein, or at multiple sites on the same protein and to other ubiquitin units, forming polyubiquitin chains on the protein [1].

**Figure 1:**
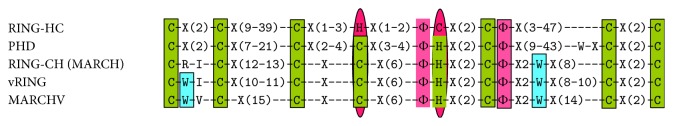
Alignment of the RING class catalytic RING domains. The figure shows the consensus alignment of six RING-HC, six PHD, all of the RING-CH (excluding MARCHV), and 11 vRING proteins. Identity is shown by the colored rectangles and red colored oval represent the identifying Cys and His residues. Additional details are given in the text. Adapted from [7].

There are currently three main classes of E3 ligases, the really interesting new gene (RING) class, the homologous to the E6-AP carboxyl terminus (HECT) class, and the RING-between-RING (RBR) class, which catalyze ubiquitin ligation through very different mechanisms (extensively reviewed in [2]). The RING E3s possess a characteristic RING finger domain that contains eight cysteine and histidine residues coordinating two Zn atoms in the interior of the protein [3]. They appear to act in as adaptors: they bind to both the substrate and E2 simultaneously, and catalyze the ligation by bringing the nucleophile on the substrate into close proximity of the E2-ubiquitin thioester bond [4]. Membrane-associated RING-CH (MARCH) proteins are RING E3s with a RING-CH domain that differs modestly from the classic RING domain (now termed RING-HC) in the identity of the fourth and fifth coordinating residues and the length of the peptide segments between the two (Figure 1, red ovals) [5]. This review will provide a brief overview of the pertinent literature regarding the MARCH proteins, and then focus on some of the particular challenges and promises associated with the study of these E3 ubiquitin ligases.

## 2. K3 and K5

K3 and K5 are RING-CH-containing E3 ligases encoded by Kaposi's sarcoma-associated herpesvirus (KSHV) that play important roles as immune evasion proteins [6]. K3 and K5 contain an N-terminal RING-CH domain followed by transmembrane domains with hydrophilic residues at the N and C terminus, suggesting a Type IV-A topology [1]. Originally, the RING-CH domain in these proteins were identified as a plant homeodomain (PHD), based on alignment (Figure 1), but were later shown to have a significantly different folding pattern, as well as having altered placement of the catalytic tryptophan that more closely resembled the RING-HC class [7]. From early on, K3 and K5 were both shown to down regulate levels of MHC I in an ubiquitination-dependent manner, an ability shared with their homolog from murid *γ*-herpesvirus 68, mK3 [8–11]. Further studies showed that K3 and K5 directly targeted MHC I for degradation via clathrin-mediated, dynamin-dependent endocytosis into early endosomes, followed by intracellular transport to the multi-vesicular body (MVB) by a Tsg101-dependent mechanism. From the MVB, the protein is targeted for lysosomal degradation via the ubiquitin-regulated ESCRT pathway [12, 13]. The RING-CH domains of viral E3 ligases of this family contain an additional tryptophan (Figure 1, light blue rectangle). Interestingly, one of the MARCH proteins, MARCH5, also encodes a tryptophan at this same location as opposed to the arginine encoded by the other cellular MARCH proteins, suggesting a possible evolutionary linkage. Solution structure modeling shows that the two tryptophan residues are located near to each other but in different orientations alongside the E2 ubiquitin-conjugating enzyme binding surface [7]. Mutation of the tryptophan to arginine in K3 or K5 results in altered ubiquitination activity on some but not all targets and in a cell type dependent manner (S. Lang and R. E. Means, unpublished data). This same mutation has yet to be explored in the context of MARCH5.

Both K3 and K5 target a wide variety of proteins, but K5 has a larger known substrate set. K3 predominately targets MHC I HLA-A, -B and -C, but it also down regulates CD1d (a ligand implicated in antigen-presentation to T-cells), PECAM (an adhesion molecule) and interferon gamma receptor 1 (IFN*γ*R1), an important cytokine receptor [14]. On the other hand, K5 down regulates MHC I HLA-A, adhesion molecules like ICAM-1, PECAM, ALCAM, and VE-cadherin, ligands for natural killer (NK) T-cells (CD1d), ligands for NK cells (MICA, MICB, AICL), IFN*γ*R1, cellular restriction factors (BST-2, also known as tetherin), the viral receptor proteins DC-SIGN (CD209) and DC-SIGNR (CD209R), the plasma membrane t-SNARE syntaxin-4 (SX4), a member of the TGF*β* family, and the ligand B7-2, which is necessary for activation of T-cells by antigen-presenting cells (APCs) [1, 10, 11, 14–22]. Additionally, K5 is able to increase signaling through a number of receptor tyrosine kinases through a ubiquitin-dependent, but otherwise unclear mechanism [21]. This wide array of targets suggests a multifaceted role for K5 in promoting immune evasion of KSHV infected cells, oncogenesis (due to induction of the Warburg effect) and viral egress, all of which have potentially critical roles in KSHV pathogenesis [19, 21–23].

Studying K3 and K5 has helped elucidate several important aspects of ubiquitin-mediated control of cellular processes, including ubiquitin conjugation on non-lysine amino acid residues. K3 and K5 can both target cysteine residues for ubiquitination via a thioester bond, while mK3 has been shown to attach ubiquitin to serine and threonine residues through an oxyester linkage [24–26].

What is more surprising than the ability of K3 and K5 to ubiquitinate a wide variety of residues is the regulatory role of the polyubiquitin chain formed on those residues by the K3 and K5 ligases. K3 and K5 are found to associate with two E2 ligases, ubc13 and ubcH5, in a stable three-protein complex where ubcH5 mono-ubiquitinates the substrate, priming it for poly-ubiquitination by ubc13 [27]. Interestingly, the polyubiquitin chains generated by K3 in this manner are linked at the Lys63 residues of the ubiquitin rather than at the canonical Lys48 position. Studies with mutant ubiquitin confirmed that Lys63-linked polyubiquitin signals are needed on MHC I in order for it to undergo endocytosis, with structural analysis showing that Lys48 ubiquitin chains adopt a closed conformation while Lys63-linked ubiquitin chains fold in a more open, extended conformation [28]. This open configuration results in exposed hydrophobic residues becoming available for binding with ubiquitin-binding domains (UBD), which may increase the affinity of the polyubiquitin chain for the UBDs on the correlating proteins involved in the endocytic pathway [1].

The topology and linkages constituting the polyubiquitin chain also play an important role in K5-mediated down regulation of MHC I. K5-generated polyubiquitin chains on MHC I are mixed chains containing both Lys63 and novel Lys11 linkages, with a putative forked chain topology; moreover, successful endocytosis of MHC I ubiquitinated by K5 requires these Lys11 and Lys63 linkages. Experiments performed with mutant ubiquitin showed a dramatic rescue of MHC I levels on the surface but no diminishment of poly-ubiquitination, suggesting that K5 can induce poly-ubiquitination at several different residues, but only polyubiquitin chains containing Lys11 and Lys63 linkages provide a signal for endocytosis [29]. Thus, endocytosis seems to be chain specific. The types of linkages and the overall conformation of the chain probably play an important role in recognition by the correct UBDs. The reason why K5 transfers ubiquitin to its substrates in these mixed chains remains to be elucidated. Two possible sources for this unusual topology are the structural constraints imposed by K5's preferential targeting of residues proximal to the membrane, and the existence of novel UBDs which preferentially bind to chains containing Lys11 linkages or mixed polyubiquitin chains [1].

## 3. The Discovery of Cellular MARCH Proteins

Through bioinformatic approaches, the MARCH proteins were identified as promising candidates for the cellular origin of viral K3 and K5 [1]. The discovery of similar E3-ubiquitin ligases in other viruses, like poxvirus and myxoma virus, provides further evidence that these viral ligases are likely derived from a common source in the host cell [6].

The first mammalian MARCH protein discovered was MARCH8, originally termed c-MIR, which was identified in human genomes using a combination of bioinformatics techniques and RT-PCR [30]. Further studies identified ten more MARCH family members for a total of eleven mammalian proteins, all possessing RING-CH domains with E3 ubiquitin ligase activity [31–33]. Four pairs of MARCH proteins, MARCH1/8, MARCH2/3, MARCH4/9, and MARCH7/10, share structural homology with each other and seem to be closely related, with preliminary work suggesting that they share similar substrates [31, 34–36]. However, the sequence homology of these pairs vary widely; more than 90% of residues in MARCH4/9's RING-CH and transmembrane domains are identical, and this is also true for MARCH1/8, but MARCH2/3 only share about 60% homology in these functional domains [37].

Generally, most MARCH proteins have an amino-terminal RING-CH domain followed by two transmembrane domains, but there are exceptions. MARCH5 has been predicted to contain four transmembrane domains, [38, 39] while MARCH6, which was identified as a mammalian homolog of the yeast E3 ligase Doa10, has up to 14 putative transmembrane domains [40, 41]. Meanwhile, MARCH7 and MARCH10 have no transmembrane domains and the RING-CH domain is closer to the C-terminal end of the protein [31, 34]. In addition to transmembrane domains, other functional domains that recur among members of the MARCH family include tyrosine-based (YXXΦ) motifs (MARCH1, 2, 3, 4, 7, 8 and 11) that may be involved in endocytosis and c-terminal PDZ-binding domains (MARCH1, 2, 3, 4, 8, 9 and 11) that have been shown to mediate protein-protein interactions in at least two members of the MARCH family [35, 36, 42].

## 4. Overview of MARCH Proteins

### 4.1. MARCH1

MARCH1 is expressed at very low levels in most tissues, however it is highly expressed in the lymph nodes and in the spleen [31]. On a cellular level it is a critical regulator of antigen presentation and is expressed in a limited number of cell types, namely immature DCs (iDCs), B cells and monocytes [43–45].

MARCH1 and its close relative, MARCH8, have been shown to play important regulatory roles in lymphocyte development. Several targets of MARCH1 have been identified: B7.2, CD98, CD95 (Fas), TfR, and MHC class II HLA-DR, -DM and -DO [6, 44, 46–50]. Overexpression of MARCH1 in HeLa cells causes CD98 to be trafficked via EEA1 positive compartments and late endosomes rather than clathrin independent endocytosis [46]. This might partially be explained by an ability of MARCH1 to interact with Bap31, a chaperone that resides mostly in the ER and assists in the export of proteins from the ER [37].

Initial studies of MARCH1 focused on its modulation of MHC II. Generation of a MARCH1 knockout (KO) mouse indicated that MHC II had a prolonged half-life in B cells compared to wild type mice due to stabilized surface expression of MHC II [50]. MHC II HLA molecules are made up of *α* and *β* subunits. MARCH1 appears to ubiquitinate one of the subunits, and also requires the presence of an endocytic motif in one of the HLA subunits for down regulation and degradation of the HLA target to occur. HLA-DM surface expression is decreased through attachment of ubiquitin to DM*α* at Lys225 and internalization, if there is a functional tyrosine based signal present in DM*β*, followed by lysosomal degradation [49]. On the other hand, HLA-DO undergoes polyubiquitination by MARCH1 and trafficking of the target requires both the dileucine and tyrosine-based endocytosis motifs in DO*β* [47].

Dendritic cells from the MARCH1 KO mouse are abnormal, as CD11c^high^ MARCH1 KO DCs have reduced surface expression of CD8 and CD4. Furthermore, LPS stimulation of MARCH1 KO DCs produce reduced amount of IL-12 and TNF-*α*. Cocultivation of OVA-stimulated irradiated MARCH1 KO DCs with naïve OT-II CD4^+^ cells leads to reduced proliferation of the CD4^+^ cells indicating that that MARCH1 KO DCs unable to present antigens. Further studies compared the MARCH1 KO DCs to DCs from MHC II knock in mice and ultimately confirmed that modulation of MHC II by MARCH1 is required for the maturation of DCs as mature DCs from both mice had similar abnormalities [51]. Further studies have shown that MARCH1 ubiquitinates peptide-loaded MHC II in immature DCs but not in mature DCs and that this leads to enhanced degradation of internalized MHC II but does not increase the rate of endocytosis [52]. More recently, it has been found that the expression of MARCH1 is transcriptionally regulated during DC maturation. As the DCs mature MARCH1 mRNA expression decreases, leading to increased MHC II and B7.2 surface expression. Expression of MARCH1 can be induced by treatment of human monocytes with LPS or IL-10 and coculture of mouse bone marrow derived macrophages that produce* Francisella tularensis* macrophage supernatant (FTMΦSN) [43, 53]. MARCH1 mRNA levels in DCs are very low in the early stages of a zymosan-induced mouse model of multiple organ dysfunction syndrome (MODS), indicating that decreased MARCH1 expression is involved in the early stages of MODS development [54]. Interestingly, IL-10 can reduce MARCH1 mRNA levels in murine splenic B cells indicating that MARCH1 transcriptional regulation occurs in a cell-type dependent context [43]. Recently, it has been shown that the ubiquitination of MHC II by MARCH1 in DCs is critical for the development of regulatory T cells (T regs) as a MARCH1 KO mice and another transgenic mouse, that expressed a mutant version of MHC II which could not be ubiquitinated (i.e. all the lysines in the C-terminus of MHC II were replaced with arginine), produced a reduced number of T regs [55].

**Figure 2:**
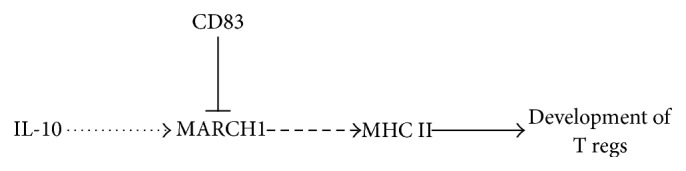
Schematic indicating the role of MARCH1 and CD83 in the development of T regs. The short dashed line indicates that IL-10 induces MARCH1 expression. The long dashed line indicates that MARCH1 targets MHC II. The solid line indicates the final outcome of MARCH1 targeting MHC II.

Expression of the CD83 transmembrane domain in DCs interferes with modulation of MHC II and B7.2 by interacting with the MARCH1 transmembrane domains, preventing MARCH1 from binding with its targets. This interference of MARCH1 by CD83 occurs in the presence of IL-10 induction, indicating that MARCH1 protein activity, as well as transcript levels, are regulated in DCs [56]. The role of MARCH1 and CD83 in the formation of T regs is summarized in Figure 2.

Tollip was identified in an shRNA screen in MelJuSo cells, a human melanoma cell line that expresses peptide loaded MHC II as well as components required for MHC II antigen presentation, as a protein involved in MHC II trafficking. Further studies on Tollip in HEK 293E CIITA cells and HeLa CIITA cells showed that knockdown of endogenous Tollip leads to decreased turnover of HLA-DR and that the cytoplasmic tail of HLA-DR are required for Tollip to act on HLA-DR. Overexpression of Tollip in the presence of overexpressed MARCH1 prevented MARCH1 from down regulating HLA-DR. This study was unable to show any interaction between MARCH1 and Tollip but did show that overexpression of Tollip decreased the expression of MARCH1. Interestingly, Tollip and MARCH1 may compete for interaction with HLA-DR as Tollip alone is able to interact with HLA-DR, but in the presence of MARCH1 this interaction is lost [57]. This indicates that there are multiple proteins that regulate the modulation of MHC II by MARCH1.

Many studies have mutated various residues within MARCH1 to determine their effect on MARCH1 modulation of its targets and itself. The half-life of murine MARCH1 is less than 0.5 h. It is degraded in lysosomes and can be stabilized through the use of cysteine protease inhibitors, specifically by inhibiting cathepsin L in APCs, but not in fibroblasts. MARCH1 contains three sorting motifs: a tyrosine based intracellular sorting motif in its N-terminus (Tyr118) and both a tyrosine-based (Tyr222) and di-leucine based (Leu215) endosomal sorting motif in its C-terminus. It was found that the di-leucine based motif was required for the full activity of MARCH1 and that the last 50 residues in the C-terminus were required for function possibly for substrate interaction or recruitment of downstream effectors leading to modulation of targets [58]. If the two endosomal sorting motifs in the C-terminus are mutated, MARCH1 is no longer incorporated into exosomes [59]. The domain between the RING-CH domain and the transmembrane domain (DIRT) is required for the activity of MARCH1; this domain was originally identified in the murid herpesvirus *γ*HV68 MARCH protein, mK3 [60]. Bioluminescence resonance transfer (BRET) has shown that altered spatial organization between the two cytoplasmic tails leads to reduced activity of the enzyme [59].

MARCH1 undergoes auto-ubiquitination or trans-ubiquitination by as yet unidentified cellular E3 ubiquitin ligases. Lys48-linked polyubiquitin chains are formed on MARCH1 followed by subsequent degradation by the proteasome. BRET assays show that MARCH1 forms homo- and heterodimers with MARCH8 and MARCH9 via its transmembrane domains. The transmembrane domains are also involved in modulation of targets, as shown through chimeras between MARCH1 and MARCH9 examining modulation of MHC I and II. These studies showed that modulation of MHC II by MARCH1 requires both the transmembrane domains of MARCH1 [61].

### 4.2. MARCH2

MARCH2 is broadly expressed in all tissues in the human body, with the highest expression being in the heart [31]. According to ONCOMINE (http://www.oncomine.org/), MARCH2 is expressed at low levels in bladder, prostate and colorectal cancers but is highly expressed in melanomas [62]. MARCH2 down regulates TfR, B7.2, DLG1 and *β*_2_-adrenergic receptor (*β*_2_AR) [31, 63, 64].

DLG1, a modular scaffolding protein that plays an important role in controlling cell polarity [65], was identified as a target that binds to the PDZ domain of MARCH2. DLG1 is normally localized at cell-cell contacts but expression of MARCH2 leads to a decrease in the amount of DLG1, as well as increased localization of DLG1 at perinuclear locations within the cell. The interaction of DLG1 and MARCH2 requires both the single PDZ domain in MARCH2 and the three domains in DLG1 [63].

*β*_2_AR is a seven transmembrane G-coupled protein receptor (GPCR). Activation of GPCRs leads to signal transductions via second messenger-mediated cellular responses. These signals are terminated when GPCRs are phosphorylated, which leads to the recruitment of *β*-arrestins and endocytosis of the receptor. Carvedilol is an antagonist of *β*_2_AR that is currently used as a *β* blocker therapy for patients with chronic high blood pressure and heart failure. It functions by recruiting MARCH2 to *β*_2_AR, leading to its ubiquitination on non-lysine residues. This causes clathrin-independent, dynamin-dependent endocytosis of the receptor, which is transported to lysosomes and degraded [64].

An affinity chromatography study of Golgi membranes enriched from a rat liver identified a number of cellular proteins that were bound to MARCH2. These were syntaxin 6 (SX6), SX8, SX13 and to a significantly lesser extent SX16, VAMP3, and VAMP4. SX6 showed the greatest affinity to MARCH2 and was shown to interact with MARCH2, whereas VAMP3 did not. SX6 is a ubiquitously expressed soluble N-ethylalemide-sensitive factor attachment protein receptor (SNARE) that is associated with the trans-Golgi network (TGN) and endosomes. SX6 may function in a number of membrane trafficking events as it forms a complex with a number of different SNAREs including Vti1a, VAMP3, and VAMP4. Overexpression of MARCH2 in COS7 cells led to the relocalization of SX6 from the periphery of the nucleus to peripheral puncta, where it colocalized with MARCH2. This was also seen for Vti1a and VAMP3, but VAMP4 only partially colocalized with MARCH2. Expression of MARCH2 in COS7 cells lead to the relocalization of proteins that are transported in both early (TGN38/46) and late endosomes (furin and mannose-6-phosphate receptor) to MARCH2 positive stained vesicles in a manner that is dependent on SX6 and therefore indicating that MARCH2 regulates the retrograde transport of proteins to the TGN. Mutation of the C-terminal PDZ domain alters the localization of MARCH2 within cells, indicating that this domain is important for MARCH2 localization [36].

One way of controlling levels of the Cystic Fibrosis transmembrane conductance regulator (CFTR) is by degrading the mature form of the protein in lysosomes through its association with CFTR-associated ligand (CAL) and SX6. MARCH2 interacts with CAL and SX6 individually, and when all three proteins are coexpressed, there is an increased interaction between MARCH2 and CAL. Overexpression of MARCH2 leads to ubiquitination and degradation of CFTR. The PDZ domain in MARCH2 is not required for interaction with CAL and is not required for degradation of CFTR, but the PDZ domain in CFTR is required for its degradation by MARCH2. This indicates that the CAL is likely the adaptor protein that directs MARCH2 to degrade CFTR [66].

### 4.3. MARCH3

MARCH3 has 63% identity to MARCH2 at the amino acid level, with the RING-CH domain of MARCH3 having 80% identity to MARCH2. MARCH3 is highly expressed in the lung, colon and spleen with low expression levels found in the brain, heart, stomach, intestine, kidneys and testis. Similar to MARCH2, cells expressing MARCH3 show increased ubiquitinylated products in the presence of the following E2 proteins: ubcH5c, ubcH6 and ubcH9. Unlike MARCH2, MARCH3 interacts with Bap31 [37].

MARCH3 also interacts with SX6 and may also be involved in the degradation of CTFR [35, 65]. Overexpression of MARCH3 leads to reduced uptake of the transferrin antibody in HeLa cells [35], although MARCH3 is unable to downregulate TfR from the cell surface [31]. MARCH3 localizes partially colocalizes with TfR, EEA1 and LAMP1 expressing vesicles indicating that it is found in early endosomes. Overexpression of MARCH3 leads to reduced cycling of TfR and a redistribution of TGN38/46 indicating that MARCH3 has a possible regulatory role in the endosomal recycling pathway. Similar to MARCH2, MARCH3 has a C-terminal PDZ domain that is required for the proper localization of MARCH3 in vesicles [35]. Interestingly, MARCH3 is unable to down regulate surface expression of targets known to be modulated by either cellular or viral MARCH proteins, such as MHC I, HLA-2.1, Fas, CD4 or B7.2, indicating that despite the homology to MARCH2 and its ability to interact with MARCH2, MARCH3 is acting in a unique way [31].

### 4.4. MARCH4

MARCH4 is approximately 62% identical to MARCH9 at the amino acid sequence level, but their RING domains are >90% identical. MARCH4 is expressed in select tissues, namely, the brain, placenta, lungs and the pancreas. It is expressed in Golgi compartments that are positive for Golgin and AP-1 (a* trans*-Golgi marker) [31]. MARCH4 interacts with Bap31 through its transmembrane domains [37]. MARCH4 utilizes ubcH2, and ubcH5A a lesser extent, as its E2 ubiquitin-conjugating enzyme [31].

Overexpression of MARCH4 leads to downregulation of surface expressed MHC I, HLA-2.1, CD4, ALCAM (a ligand for CD6), Mult1 (a ligand for NKG2D), SX4 (a SNARE), CD81 (a tetraspanin), and B7.2 to a much lesser extent [15, 31, 37, 67].

To determine which regions of MARCH4 are required to downregulate the surface expression of MHC I, truncated forms of MARCH4 were created. When either the N-terminus preceding the RING-CH domain or the C-terminus after the transmembrane domains were deleted MHC I was still downregulated, but if both regions were removed this construction was unable to downregulate surface expression of MHC I, indicating that these regions were not necessary for normal localization or function as seen for the other MARCH proteins. MARCH4 decreases surface levels of MHC I through endocytosis of the protein from the cell surface and is then sorted by the MVB pathway. MARCH4 monoubiquitinates MHC I. MARCH4 requires lysines to be present in the C-terminus of HLA-2.1 and CD4 for it to downregulate their surface expression [31].

Mult1 is a ligand for the NKG2D receptor expressed on NK cells. Coexpression of Mult1 and MARCH4 leads to decreased surface expression of Mult1. MARCH4 is able to ubiquitinate and interact with Mult1 through the C-terminus of Mult1, even when all the lysines in the C-terminus are mutated to arginine indicating that MARCH4 is able to ubiquitinate Mult1 on residues other than lysines. Expression of murine MARCH4 in C1498 cells reduced their susceptibility to cell lysis when cocultured with NK cells, to a lesser extent than when MARCH9 is expressed in these cells, as it does not reduce Mult1 surface expression to the same extent as MARCH9 does [67].

CD81 is a tetraspanin, which is degraded via the lysosomes when MARCH4 is expressed in human foreskin fibroblasts. CD81 is localized to LAMP1 expressing vesicles in the presence of MARCH4. siRNA knockdown of endogenous MARCH4 in human foreskin fibroblasts leads to an increase in CD81 surface levels, indicating that CD81 is a* bona fide* target of MARCH4 [37].

SX4 is a target-membrane associated-SNARE that is found in the plasma membrane and mediates the docking of transport vesicles to the cell surface. In the presence of MARCH4, SX4 has reduced surface expression and is localized to the Golgi of HeLa cells. SX4 is somewhat of an unusual target for MARCH proteins as it is a type II transmembrane protein. With MARCH4 targeting SX4, this adds to a growing body of data that points towards the MARCH proteins altering the movement of proteins through MVB pathway and could indicate that the MARCH proteins are altering exocytosis as well [15].

### 4.5. MARCH5

MARCH5, also known as MITOL (mitochondrial ubiquitin ligase), has four transmembrane domains and is found in the mitochondrial outer membrane (MOM), with the RING domain exposed to the cytoplasm. Northern blot analysis shows that MARCH5 is expressed in all tissues; this was confirmed by a large transcriptome study that also indicates that MARCH5 is highly expressed in bronchial epithelial cells, the thyroid, and B cells [38, 68]. Studies have shown that it plays an important role in regulating mitochondrial morphology. Mitochondria frequently fuse and divide to form dynamic networks in eukaryotic cells, and the key molecules in this process are mitofusins 1 and 2 (MFN1 and MFN2), which are large transmembrane GTPases, necessary for fusion. Division of mitochondrial is controlled by the mitochondrial fission protein, hFis1, which is located in the MOM, and Drp1, which a cytosolic dynamin-related GTPase [38, 39]. MARCH5 autoubiquitinates itself and its E3 ubiquitin ligase activity is required to prevent accumulation of fragmented mitochondria [38].

In HeLa cells stably expressing MARCH5 it interacts with MFN2, ubiquitinated hFis1 and ubiquitinated Drp1 [38, 39]. Overexpression of MARCH5 in COS7 cells leads to the formation of elongated mitochondria, which was overcome by coexpressing a dominant negative mutant of MFN2 that lacked the transmembrane domains. According to two studies, mitochondrial fragmentation in HeLa cells following expression of a MARCH5 RING domain mutant, generated through either a double point mutation in the coordinating cysteine residues (C65S and C68S) or a single point mutation of a catalytic tryptophan (H43W) can be overcome by expressing a dominant negative mutant of Drp1 (Drp1 K38A, which is GTPase deficient). RING mutants of MARCH5 colocalize with Drp1 and MFN2 but not hFis1. This indicates that MARCH5 acts upstream of Drp1. The expression of MARCH5 leads to ubiquitination of hFis1 and the subsequent decreased expression of hFis1. Overall this indicates that while MARCH5 influences the cellular location of Drp1 and MFN2, MARCH5 does not alter hFis1 localization but instead leads to it degradation. All of these activities are reliant on the ubiquitin ligase activity of MARCH5 [38, 69]. On the other hand, another group examined the protein levels of hFis1, Drp1, and MFN2 in HeLa cells in which MARCH5 had been knocked down and found that there was no change in these proteins, but there was an increase in MFN1 protein levels. This study showed that decreased MARCH5 expression leads to increased mitochondrial mass, increased intracellular reactive oxygen species (ROS), decreased mitochondrial membrane potential, decreased ATP production, decreased mitochondrial DNA, and reduced proliferation, which were all signs of stress-induced senescence. MARCH5 and MFN1 interacted leading to the proteasomal degradation. There is some discrepancy over whether MARCH5 interacts with MFN1, according to a study performed by overexpressing MFN1 in HeLa cells, Park et al. showed that MARCH5 did interact with MFN1 whereas Sugiura et al. overexpressed MFN1 and MARCH5 in HEK 293 cells and showed that the two proteins were not interacting [70, 71]. The study by Park et al. showed that the expression of dominant-negative MFN1 (T109A that is GTPase deficient) in HeLa cells knocked down for MARCH5 expression led to reduced senescence, whereas the expression of dominant-negative Drp1 (K38A) had no effect [71]. This agreed with the previous studies indicating that MARCH5 acted upstream of Drp1.

During the cell cycle, mitochondrial fragmentation occurs during prophase of mitosis through phosphorylation of Drp1 by Cdk1/cyclin B1. Subsequently, the completion of the development of the mitochondria occurs during the G_2_/M phase. MFN1 contains a putative cyclin B1 phosphorylation site and interacts with cyclin B1 in asynchronously dividing cells. This interaction was increased during G_2_/M and was further increased by the presence of a proteasome inhibitor (MG132). Increased polyubiquitinated MFN1 is found in G_2_/M phase cells expressing MARCH5 and treated with MG132. This study indicates that MARCH5 may be involved in regulating MFN1 during the cell cycle leading to the regulation of mitochondrial networks [72]. Further studies will be required to prove the relationship between MFN1 and cyclin B1 and MARCH5.

MFN2 is mostly found at mitochondrial-associated ER membrane (MAM). Interestingly MARCH5 is only able to interact with MFN2 associated with the mitochondria but not the ER. MFN2 contains an N-terminal heptad repeat region (HR1) that is required to interact with the C-terminus of MARCH5. MARCH5 uses ubcH5b to ubiquitinate MFN2 leading to the addition of Lys63-linked polyubiquitin chains. Reduced expression of MARCH5 in mouse embryonic fibroblasts (MEFs) leads to decreased MFN2 in the MAM, decreased ER-mitochondrial colocalization due to reduced tethering of the ER to the mitochondria and decreased mitochondrial calcium uptake. This indicates that by ubiquitinating MFN2, MARCH5 regulates the formation and the function of the MAM. Mitochondrial-ER bridges are formed due to oligomerization of MFN2 in a GTP-dependent manner. MARCH5 is required to initiate MFN2 oligomerization through ubiquitination of lysine 192, which is located in the GTPase domain of MFN2 [70].

MARCH5 appears to reduce the presence of mis-folded proteins in the mitochondria. Familial amyotrophic lateral sclerosis (ALS) occurs due to the accumulation of mutant superoxide dismutase 1 (mSOD1) in neuronal cells. mSOD1 accumulates in the mitochondria leading to reduced reactive oxygen species (ROS) scavenging activity. mSOD1 partially colocalizes with MARCH5 in the mitochondria. Interestingly, MARCH5 could only interact with mSOD1 not wild type SOD1, and this interaction led to enhanced ubiquitination of mSOD1, in a manner that depended on the E3 ubiquitin ligase activity of MARCH5. MARCH5 utilizes ubcH5a, ubcH5b, or ubcH5c for this ubiquitination. Ubiquitination of mSOD1 by MARCH5 leads to its degradation via the proteasome thus reducing the toxicity of mSOD1 accumulation, thus leading to reduced cell death and reduced ROS generation. Overall this points to MARCH5 having a protective role in the mitochondria preventing the accumulation of toxic proteins such as mSOD1 or mutant short chain acyl CoA dehydrogenase [73].

Similarly, the expansion of glutamine residues in ataxin-3 leads to Machado-Joseph disease, which is a neurodegenerative disorder that occurs due to the accumulation of this toxic protein in the nucleus and the mitochondria. One model for studying this disease is the use of an N-terminal truncated ataxin-3 that has 71 glutamines (ΔNAT-3Q71). ΔNAT-3Q71 partially localizes to the mitochondria and interacts with MARCH5, independent of the ubiquitin ligase activity of MARCH5. Mitochondrial ΔNAT-3Q71 is ubiquitinated using ubcH5b and is degraded by the proteasome. Expression of MARCH5 leads to a reduction in insoluble ΔNAT-3Q71 present, reduced cell death due to ΔNAT-3Q71 expression, increased ATP production and reduced ΔNAT-3Q71-induced cytochrome* c *(an apoptosis inducing factor) release, indicating overall that MARCH5 prevents cells from accumulating toxic proteins in its mitochondria [74].

Microtubule-associated protein 1B (MAP1B) is made up of a heavy chain and a light chain (LC1) and has an important role in the stability of the cytoskeleton. LC1 has been implicated in a number of neurological disorders including fragile-X syndrome and Parkinson's disease. Nitric oxide (NO) is an important signaling molecule, which when present in excess leads to a number of neurodegenerative diseases. NO signals are propagated through the S-nitrosylation of proteins, in which a nitrogen monoxide group is covalently attached to the thiol side chain of a cysteine residue. LC1 undergoes S-nitrosylation on Cys257, leading to a conformational change and subsequent translocation to microtubules. Through a yeast-two-hybrid screen on mouse brain cDNA library using a C-terminal region of MARCH5 (amino acids 257–278), MAP1B-LC1 was identified as MARCH5 binding partner. LC1 is located in the cytosol and in the mitochondria, specifically on the surface of the outer mitochondrial membrane but is not embedded in the membrane. Endogenous MARCH5 interacts with LC1 through a conserved region (amino acids 195–215) in LC1 and this interaction occurs independent of whether LC1 undergoes S-nitrosylation. This conserved region and S-nitrosylation are required for ubiquitination of LC1 by MARCH5, which is induced in an NO-dependent manner. LC1 is made up of a microtubule-binding site (MTB), a mitochondrial aggregation, and genome destruction (MAGD) domain and an actin-binding site (AB), which contains the ubiquitin binding sites. Increases in intracellular calcium lead to nNOS activation and thus subsequent S-nitrosylation of LC1. In the absence of calcium, the ubiquitination sites in AB are masked by MTB, and it is only upon increases in intracellular calcium that S-nitrosylation occurs leading to unmasking of this region for MARCH5 to be able to ubiquitinate LC1, thus indicating that a conformational change in LC1 needs to occur before MARCH5 is able to induce ubiquitin-dependent degradation of mitochondrial LC1. Mitochondrial S-nitrosylated LC1 is degraded via the proteasome in the presence of MARCH5, which prevents LC1 from aggregating in the mitochondria. Overall, the degradation of S-nitrosylated LC1 by MARCH5 enabled cortical neurons to undergo radial migration as well as increased their cell viability, thus blocking LC1-mediated cytotoxicity. Under conditions of excess NO production, MARCH5 can undergo S-nitrosylation, which inactivates MARCH5. Therefore while NO production induces MARCH5 protective functions on the mitochondria by leading to ubiquitination and subsequent degradation of LC1, excess NO can lead to MARCH5 dysfunction [75].

In line with MARCH5 playing a protective role in the cells, it also has a role in innate immunity through positively regulating signaling through Toll-like Receptor 7 (TLR7), which senses the presence of viral single stranded RNA. When activated, TLR7 triggers the recruitment of MyD88, which in turn recruits IRAK1 and IRAK4. IRAK4 phosphorylates IRAK1 leading to their dissociation from MyD88 and subsequent interaction with TRAF6, which is an E3 ubiquitin ligase. Ubc13 and Uev1A help catalyze the formation of Lys63 polyubiquitin chains serving as a basis for a protein complex that includes TRAF3, IKK*α*, and IRF7. This complex leads to the induction of type I interferons (IFNs) and interferon-inducible genes (ISGs). TANK (also known as I-TRAF) is a negative regulator of TLR-mediated proinflammatory cytokines, such as IL6 and TNF*α*, but not ISGs, such as ISG15, through the suppression of TRAF6. MARCH5 is able to interact with TRAF6, MAVS, and TANK but is only able to ubiquitinate TANK, utilizing lysine residues in the C-terminus of TANK. MARCH5 generates Lys63-linked polyubiquitin chains on TANK. Interaction between MARCH5 and TANK requires the C-terminus of TANK and does not require the transmembrane domains of MARCH5. The interaction between MARCH5 and TANK is enhanced in cells treated with R837 (also known as Imiquimod), which is a TLR7 ligand. TRAF6 is ubiquitinated in the presence of MARCH5 in a manner that is dependent on TANK being absent, indicating that MARCH5 regulates TRAF6 autoubiquitination by relieving the inhibitory presence of TANK by ubiquitinating TANK leading to the degradation of TANK [76].

Further evidence of MARCH5 playing a role in stress response has been shown through studying the death of retinal ganglion cells, which in glaucoma patients leads to loss of vision. These mitochondria in these cells undergo fragmentation when subjected to conditions of elevated pressure, oxidative stress, or ischemia-reperfusion. This can be reversed through the expression of RING mutant MARCH5 (H43W) or dominant-negative Drp1 (K38A) [77]. Similarly when neuroblastoma cells undergo neurodegenerative stress, the mitochondrial potential in cells expressing the RING mutant of MARCH5 (H43W) is the same as unstressed cells expressing wild type MARCH5 [78]. This indicates that while MARCH5 does not protect neuronal cells from stress it does play a role in the decision to undergo stress-induced mitochondrial fragmentation.

**Figure 3:**
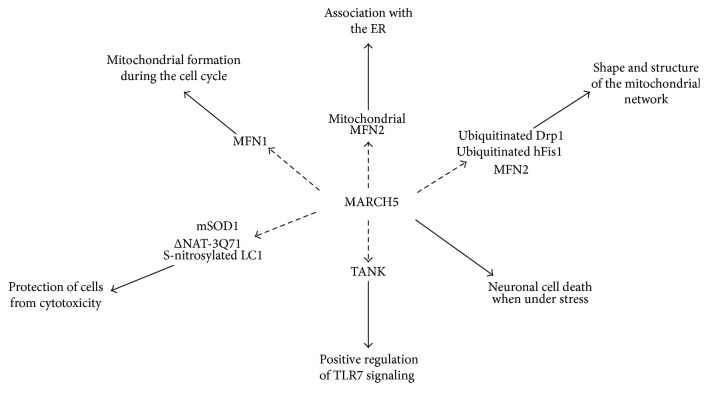
Summary of the known targets and function of MARCH5. The dashed lines indicate the proteins that MARCH5 targets. The solid lines indicate the final outcome.

Overall, MARCH5 has multiple functions in the mitochondria, with the major functions summarized in Figure 3. It regulates mitochondrial fission and fusion as well as MAM formation. MARCH5 has a protective role through causing the degradation of misfolded proteins that would otherwise accumulate in the mitochondria as well as positively regulating signaling through TLR7. Interestingly, while MARCH5 can protect cells from undergoing senescence it is also involved in the decision for neuronal cells to undergo stress-induced mitochondrial fragmentation leading to cell death.

### 4.6. MARCH6

MARCH6 was first identified as a TEB4, a mammalian ortholog of the yeast transmembrane protein Doa10, an E3 ligase located in the membrane of the ER and nuclear envelope that is implicated in endoplasmic reticulum-associated degradation (ERAD) [40, 41]. Interestingly, the* MARCH6* gene is located in the region that is associated with Cri-du-chat syndrome, a neurodegenerative disorder [41].

In humans, MARCH6 is expressed in the following tissues: the heart, brain, placenta, lung, liver, skeletal muscle, kidney, pancreas, thymus, prostate, testis, ovary, small intestine, and spleen (in which its expression is the lowest). It is expressed at very low levels in the liver in rats but is not expressed in the brown adipose tissue or the thyroid [79, 80]. The expression of MARCH6 is not altered upon ER-induced stress [41].

Several features of MARCH6 make it unusual compared to the other family members. The first is its size: the 910-residue protein is the largest of the MARCH family members, with 14 putative transmembrane domains and a 130aa region called the TEB4-Doa (TD) domain that is conserved across a broad range of eukaryotic species, from mouse to* Arabidopsis* [40, 41]. Like its yeast homolog, Doa10, the RING-CH domain and the C-terminus are located in the cytosol. Similarly, the RING-CH domain of MARCH6 has been shown to possess the ability to ubiquitinate and degrade itself in* in vitro* ubiquitination assays with the E2 ligase ubc7. MARCH6 forms Lys48-linked ubiquitin chains, which is company linked to proteasomal degradation. Ubc7 is also involved in ERAD and is also typically found associated with the ER membrane, providing further evidence that MARCH6 is an ER membrane protein that plays an important role in ERAD [40, 41].

The thyroid hormone activating type 2 iodothyronine deiodinase (D2) is a key enzyme that regulates 3,5,3′-triiodothryonine (T3) generation, which is important when humans are exposed to the cold. MARCH6 interacts with ubiquitinates and degrades D2 [80]. While this study indicated that MARCH6 was involved in degradation of D2, it did not show that D2 was being degraded by ERAD.

Studies performed examining the difference in steady state protein levels between* doa10Δ* mutant yeast compared to wild type yeast identified Erg1 as a novel substrate of Doa10. Erg1 is a squalene monooxygenase that is a component of the mevalonate pathway, which regulates sterol levels in the ER, in* Saccharomyces cerevisiae*. The degradation of Erg1 by Doa10 does not involve proteins that are involved in degrading misfolded proteins but does involve proteins that are involved in ERAD, specifically Cdc48, which is an ATPase involved in extruding proteins from the ER membrane. This modulation of Erg1 by Doa10 is important in dictating membrane fluidity. The mammalian homolog of Erg1 is SM. SM is ubiquitinated and degraded by the proteasome due to production of cholesterol (the end product of the pathway rather than an intermediate product). Due to the similarities between Erg1 and SM, it was not unsurprising that SM is a target of MARCH6 and that SM is degraded due to increased amounts of cholesterol. This indicates that ERAD may not only be important in degrading misfolded proteins but also in homeostasis of functional proteins [81].

Progressive familial intrahepatic cholestasis type II (PFIC II) occurs due to a number of different mutations in the* BSEP* gene, which encodes an ATP-binding cassette transporter involved in the salt export pump for the canalicular excretion of bile salts. The mutations that arise in PFIC II patients are premature terminations, missense, and frame shift mutations that lead to canalicular mislocalization and/or impaired transport function of Bsep, whereas some of these mutations lead to reduced protein levels of Bsep, but not mRNA levels, indicating that the protein was being degraded. Wang et al. studied mutants of rat Bsep that showed reduced protein levels and altered glycosylation, that is, they were either not glycosylated or contained immature glycans, and showed that these mutants showed increased ubiquitination in the presence of a proteasome inhibitor. Furthermore these mutants were mostly localized to the ER, whereas wild type Bsep, which has mature glycans, is localized at the cell surface. The authors showed that the nonglycosylated Bsep underwent ERAD via Hrd1, but other immature glycosylated mutants were undergoing ERAD via MARCH6. This indicates that MARCH6 is involved in ERAD and potentially recognizes different misfolded substrates compared to Hrd1. The mutants that were targeted by MARCH6 had their mutations located on the cytoplasmic side of the ER, whereas the mutant targeted by Hrd1 had its mutation located on the luminal side of the ER. This led Wang et al. to propose that the location of the mutations determines the target specificity of the ERAD E3 ubiquitin ligase [82].

### 4.7. MARCH7

MARCH7, also known as axotrophin (axot), is unusual because it encodes a RING-CH domain but no predicted transmembrane domains. The N-terminus of MARCH7 contains a disordered but serine/proline rich region with the RING-CH domain being found closer to the C-terminus [83, 84].

MARCH7 was one of 216 genes found to be enriched in mouse embryonic, neural and hematopoietic stem cells and thus considered to be a core gene conferring stem cell properties (also referred to as “stemness”) [85]. Studies in mice showed that MARCH7 mRNA is highly expressed in most developing mouse tissues up to E15.5 with the highest expression levels being found in the nervous system. In adult mice MARCH7 is mostly expressed in the brain, thymus, muscle, and kidney, but very little is expressed in the spleen or liver. It should be noted that Metcalfe et al. do not show this data but summarize this from unpublished data from Haendel and Lyons [86]. On the other hand, a study by Szigyarto et al. performed an indepth analysis of MARCH7 expression in 48 human tissue types and showed that epithelial cells and trophoblasts demonstrated the greatest expression. The majority of tissue types examined showed positive expression, although in some tissues certain cell types showed differential expression, for example, in the cerebellum neurons were 100% positive for MARCH7 expression whereas glial cells did not express MARCH7. While in the lymph nodes, lymphocytes were positive for MARCH7 expression but B cells were not [87]. With MARCH7 being highly expressed in stem cells, neurons, and lymphocytes, this suggested a multifaceted role in early development and the immune system [85].

Recent immunohistochemical studies in tissues from rat testes showed that MARCH7 is highly expressed in developing rat spermatids, colocalizing with *β*-actin. MARCH7 is highly expressed in the head of the spermatid and at very low levels in the flagellum, but as the developing spermatid elongates, MARCH7 expression extends along the flagellum until it is expressed along its whole length, thus indicating that MARCH7 may also play a role in spermatogenesis [88].

Like many MARCH proteins, MARCH7 ubiquitinates itself possibly utilizing Huntingtin-interacting protein 2 and ubc13 as E2 conjugating enzymes [83]. Studies examining the polyubiquitination of newly synthesized MHC class I heavy chains (HCs) in the presence of US11, a protein expressed by the human cytomegalovirus for transport of the HCs from the ER to the cytosol, identified E2–25K as the E2 ubiquitin-conjugating enzyme that was required for this reaction. The RING domain from MARCH7, but not MARCH1 or MARCH6 could catalyze the ubiquitination reaction, thus indicating that E2–25K is potentially another MARCH7-interacting E2 conjugating enzyme [89].

MARCH7 also interacts with two deubiquitinating enzymes (DUBs): ubiquitin-specific protease (USP)9X and USP7, which are located in the cytosol and nucleus, respectively. MARCH7 is predominantly localized in the nucleus of cells, with some seen in the cytoplasm. A RING-CH mutant of MARCH7 (MARCH7 W589A/I556A) is found predominantly in the plasma membrane with some expression seen in the nucleus, indicating that MARCH7 traffics between the nucleus and the plasma membrane. The expression of USP9X and USP7 stabilizes MARCH7 in either the cytosol or the nucleus respectively [83]. MARCH7 encodes a nonclassical importin-*α* nuclear localization signal (SKRPKL), which dictates its nuclear localization as mutation of both lysine residues leads to the cytoplasmic localization of MARCH7. The C-terminus of MARCH7 may also encode a leucine rich nuclear export signal but this is yet to be tested experimentally [12].

MARCH7 KO mice are viable and fertile. The only easily distinguishable phenotype is impaired development in their nervous systems, with notable agenesis of the corpus callosum and early axonal degeneration of dorsal root ganglia. This indicates that MARCH7 may have a role in neuronal development [86]. Supporting this observation, MARCH7 appears to be preferentially expressed in cells of neuronal origin [83].

B cells develop normally in MARCH7 KO mice show. On the other hand, the T cells undergo hyperproliferation after stimulus with concanavalin A and produce increased IL2 and leukemia inhibitory factor (LIF) but not interferon *γ* (IFN*γ*) or IL4. Expression of LIF is connected to immune tolerance, whereas IFN*γ* expression is linked to rejection indicating that MARCH7 played a critical role in regulating immune tolerance [86]. A subtractive gene array comparing splenocytes from a mouse model in which tolerance can be generated to a mismatched graft versus splenocytes from a mouse that rejected the graft identified MARCH7 as one of 8 genes implicated in immune tolerance [90]. Forkhead transcription factor P3 (Foxp3) is a central regulator of immune self-tolerance; its expression directs CD4^+^ naïve T cells to a T reg cell fate. Thymic cells from MARCH7 KO mice have reduced expression of Foxp3 [91]. Expression of Foxp3 expression induces MARCH7 expression, indicating that these genes tightly regulate one another. Grafting a mismatched tissue onto MARCH7 KO mouse leads to increased graft survival, which is attributed to the upregulation of LIF, and splenomegaly, indicating that MARCH7 downregulates the activated T cell response [92]. While Foxp3 expression directs T cells to a T reg cell fate, IL6 directs cells to a Th17 lineage. IL6 and LIF belong to the IL6 family of cytokines but their expression opposes one another. The LIF receptor is a heterodimer of gp190 and gp130, whereas the IL6 receptor is a homodimer of gp130. The gp190 subunit is regulated in two ways: (1) IL6 represses gp190 expression and (2) MARCH7 may degrade gp190 once it is expressed in activated T cells. Further evidence needs to be presented to confirm that gp190 is a target of MARCH7 but if it were then this would provide evidence that MARCH7 assists in directing naïve T cells to develop into Th17 cells [93].

### 4.8. MARCH8

**Figure 4:**
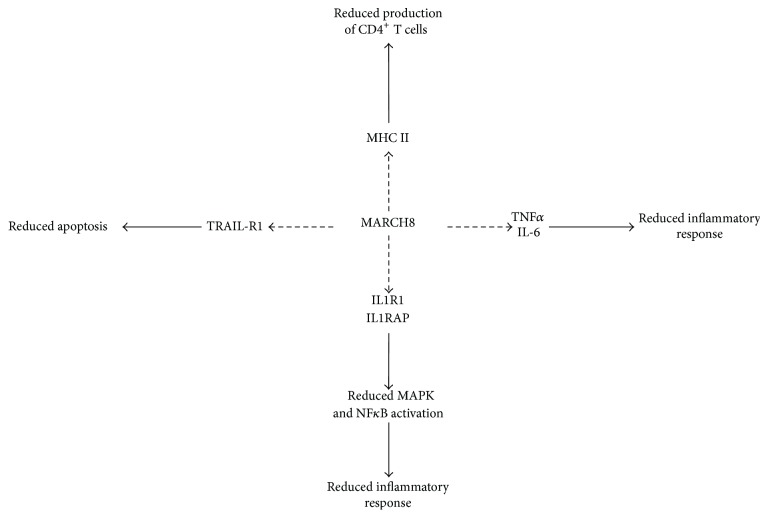
Summary of the known targets and functions of MARCH8. The dashed lines indicate the proteins that MARCH8 targets. The solid lines indicate the final outcome.

As mentioned earlier, MARCH8 was the first cellular MARCH E3 ligase to be characterized and plays a number of roles in the immune response, as summarized in Figure 4 [30]. It has two transmembrane domains with the RING-CH domain found on the cytosolic side and two putative tyrosine based endocytic motifs found in the C-terminus [34]. MARCH8 is expressed in the following human tissues and cell types: neonatal brain, lymph node, spleen, placenta, heart, liver, kidney, lung (highest expression), muscle (lowest expression), pancreas, thymus (very low expression), tonsil, fetal liver, bone marrow, B cells, monocytes, and dendritic cells. Immunofluorescence studies show that MARCH8 was found in early endosomes, late endosomes, and at the cell surface [30, 31, 94].

MARCH8 autoubiquitinates itself and utilizes ubcH2 and ubcH5a as E2 ubiquitin-conjugating enzymes [30, 31]. Overexpression of MARCH8 leads to the downregulation of several immunomodulatory receptors, including MHC I HLA 2.1, MHC II, CD95 (Fas), B7.2, TfR, CD166, CD44, CD88, and CD98, indicating that it plays a significant role in immune suppression [6, 15, 30, 31, 37, 46, 95, 96]. In support of this hypothesis, transgenic mice that express MARCH8 under an invariant chain promoter and express high levels of MARCH8 in APCs are resistant to the onset of experimental autoimmune encephalomyelitis. Furthermore DCs from these mice are unable to stimulate T cells. These transgenic mice produce half as many CD4^+^ T cells in the thymus compared to control littermates, but produce the same number of CD8^+^ T cells. This result led to the identification of MHC II as target of MARCH8 [95].

Many studies have been performed on different isotypes of MHC II to determine which residues are important for the regulation by MARCH8, although it should be pointed out that downregulation of MHC II has not been examined in a knockout mouse model. Mature MHC II occurs as a heterodimer of *α* and *β* chains. The *β* chain of I-A^d^ has a single lysine residue (K225) and this lysine is the target of ubiquitination by MARCH8 leading to increased endocytosis and degradation of the *β* chain [95]. Unlike I-A^d^, both of the *α* and *β* chains of DR are subject to ubiquitination by MARCH8, and interestingly mutation of K225 in DR*β* does not prevent reduced surface HLA-DR expression in the presence of MARCH8 [94]. Further studies have shown that the exact position of the lysine in DR*β* is important as K225 within one amino acid from the transmembrane domain prevented MARCH8 from downregulating and ubiquitinating DR*β*, but MARCH8 was still able to downregulate DR*β* when the K225 is moved between 2 and 7 amino acids away from the transmembrane domain. Other sequences surrounding the lysine may also play a role in regulation of DR*β* by MARCH8. For example, ^234^GLLS^237^ may serve as a dileucine-motif for endocytic trafficking and the amino acids in the extracellular portion of DR*β* proximal to the transmembrane region need to be either positively or negatively charged for downregulation to efficiently occur [97]. K219 of DR*α* is subject to ubiquitination by MARCH8, although ubiquitination of DR*β* appears to be dominant and more effective at reducing surface expression of HLA-DR. This is likely due to sequences surrounding K219 in DR*α* as mutations of the surrounding sequences to alanine lead to increased downregulation of HLA-DR [94]. In studies looking at the impact of MARCH8-driven ubiquitination on HLA-DR it was found that MARCH8 was able to redirect HLA-DR from exosomes into a degradatory pathway in a cell-type dependent manner [98]. MARCH8 causes greater ubiquitination of HLA-DO, compared to MARCH1 or MARCH9, even though MARCH9 causes greater surface modulation of HLA-DO [47]. MARCH8 is able to ubiquitinate HLA-DM*α* alone on residue K230, leading to its downregulation and degradation. MARCH8 induced modulation of HLA-DM dimer requires the tyrosine based signal on DM*β*, but does not require ubiquitination of DM*α* [49].

Given that overexpression of MARCH8 leads to the impaired development of CD4 T^+^ cells, it is not surprising that MARCH8 transgenic mice are resistant to developing arthritis when injected with collagen type 2 (CII). Overexpression of MARCH8 in DCs of a CII-induced arthritis mouse model leads to reduced arthritis as well as TNF*α* and IL6 expression in synovial tissues, indicating that expression of MARCH8 alters the local inflammatory immune response but not the systemic immune response. This is due to the reduced production of inflammatory cytokines. This implies a new role for MARCH8 in inflammation and as a potential therapeutic treatment for arthritis [99].

Along the same lines, overexpression of MARCH8 in HEK 293 cells stimulated with IL-1*β* leads to the downregulation of IL-1 receptor accessory protein (IL1RAP) and thus prevents subsequent activation of NF*κ*B. This is dependent on the E3 ubiquitin ligase activity of MARCH8. IL1RAP is part of a membrane bound receptor complex with IL-1 receptor type 1 (IL1R1) that recognizes the proinflammatory cytokine IL-1*β*. Recognition of IL-1*β* by the receptor complex leads to recruitment of intracellular adaptor proteins and kinases and eventual activation of NF*κ*B. MARCH8 interacts in pull-down experiments with both IL1R1 and IL1RAP, but only decreases the protein levels of IL1RAP. The interaction between MARCH8 and IL1RAP requires the transmembrane domain of IL1RAP and the first transmembrane domain and the region between the two transmembrane domains of MARCH8. Immunofluorescence studies show that IL1RAP and MARCH8 colocalize. The overexpression of MARCH8 and IL1RAP leads to Lys48-linked polyubiquitination of IL1RAP on K512 in the C-terminus of IL1RAP with ubc5B/C serving as the E2 ubiquitin conjugating enzyme. IL-1*β* stimulation of cells leads to the activation of the MAPK pathway, and overexpression of MARCH8 negatively regulates this pathway. Overall this indicates that MARCH8 plays a significant role in attenuating the inflammatory immune response [100].

MARCH8 has also been shown to downregulate TNF-related apoptosis inducing ligand receptor 1 (TRAIL-R1; also known as DR4), but not TRAIL-R2, from the surface of breast cancer cells. Upon recognition of TRAIL ligands expressed on NK cells, a signaling cascade is induced, generally through the NF*κ*B pathway, ultimately leading to apoptosis. MARCH8 causes increased endocytosis of TRAIL-R1 in a dynamin dependent manner. MARCH8 changes the trafficking of TRAIL-R1 from recycling endosomes to lysosomes, where it is degraded. Endogenous MARCH8 ubiquitinates K273 located in the C-terminus of TRAIL-R1. Overall the expression of MARCH8 prevents cells from undergoing apoptosis indicating that targeting MARCH8 for knockdown may be of therapeutic benefit to patients with cancer [101].

A stable isotope labeling of amino acids (SILAC) experiment was performed to identify novel targets of K5 in HeLa cells leading to the identification of syntaxin 4 (SX4) and CD166, which were subsequently also shown to be targets of MARCH8. SX4 is a member of the transmembrane-associated soluble N-ethylmalemidine fusion attachment protein receptor family and is involved in the docking of transport vesicles at the cell surface. Expression of MARCH8 in HeLa cells leads to the relocalization of SX4 from the plasma membrane to the Golgi and endosomes as well as an overall reduction in SX4 protein levels. Deletion studies of the C-terminus of MARCH8 showed that a sequence that is found between Δ46 and Δ62 truncation mutants is required for MARCH8 to exit the ER and target SX4 for relocalization. On the other hand, CD166 is a type I transmembrane glycoprotein that is a ligand for CD6 on T cells. The interaction between CD166 and CD6 is part of the immunological synapse. MARCH8 is able to partially downregulate surface expression of CD166 but to a lesser extent than MARCH4 or MARCH9. MARCH8 can ubiquitinate CD166 but to a significantly lesser extent than K5 [15].

Another SILAC performed on primary human foreskin fibroblasts overexpressing MARCH8 led to the discovery of 11 additional potential targets of MARCH8, in addition to the already established CD166. In this study, the authors focused on CD44, CD81, and Bap31. CD44 is a type I transmembrane glycoprotein that is involved in cell-to-cell contacts and acts as a receptor for a number of proteins including hyaluronic acid. CD81 is a tetraspanin that functions as part of different complexes, depending on the cell type, to enable cells to recognize different stimuli. CD44 and CD81 are degraded via lysosomes when MARCH8 is overexpressed. Despite this data, neither CD44 nor CD81 surface expression increased upon MARCH8 knockdown in human foreskin fibroblasts; this maybe because this cell type does not endogenously express high levels of MARCH8 and therefore MARCH8 expression may not influence these targets over MARCH4, which is expressed at much higher levels in this cell line and has been shown to modulate these targets. To verify whether CD44 and CD81 are targets of MARCH8 it may be necessary to perform knockdown experiments in cells that express higher endogenous levels of MARCH8, such as immature dendritic cells. Surprisingly, MARCH1, the MARCH protein that has the greatest homology to MARCH8, is unable to modulate CD44 or CD81 [37].

CD44 and CD98 are normally trafficked via a clathrin independent endocytosis pathway, whereby rather than being sorted into early endosomal antigen 1 (EEA1) positive endosome, they enter the tubular recycling endosomes directly. In the presence of MARCH8, these proteins still undergo clathrin independent endocytosis but their trafficking is altered to EEA1 positive endosomes followed by subsequent degradation in late endosomes. TSG101, a component of the ESCRT-1 complex, is found to be necessary for trafficking of CD44 and CD98 in the presence of MARCH8. CD98 is ubiquitinated in the presence of MARCH8 [46].

Bap31 is a chaperone that resides in the ER membrane and is involved in the movement of transmembrane proteins from the ER to the Golgi as well as caspase-8 mediated apoptosis. MARCH8 reduces the surface expression of a small portion of Bap31 that normally localizes to the cell surface but does not appear to affect the total amounts of Bap31 in the cell. MARCH8 interacts with Bap31 through the transmembrane domains of Bap31, which enables the proper folding, assembly and intracellular transport of MARCH8 [37].

Surprisingly, MARCH8 has been identified in an siRNA screen aimed to find restriction factors to HIV-1 replication [102]. Further experimentation is required to determine the step at which MARCH8 inhibits HIV-1 replication, however this does indicate that this MARCH protein has a role in protecting cells from invading pathogens.

The TfR is a type II membrane protein that is involved in regulating the uptake of Tf-bound iron from the plasma into cells. The mRNA stability of TfR is one mechanism involved in regulating its expression. The C-terminus contains a canonical tyrosine based endocytosis motif. Overexpression of MARCH8 leads to the downregulation of surface TfR indicating that this is another method of regulating surface TfR [31]. Further study has shown that TfR and MARCH8 interact with one another through the transmembrane and cytoplasmic domains of TfR, but only the transmembrane domain is required to be present for MARCH8 to downregulate surface expression of TfR. Amino acids 222–231 in the C-terminus of MARCH8 are required to interact with TfR. MARCH8 is able to ubiquitinate TfR on lysine residues in the C-terminus leading to its degradation in lysosomes. Six amino acids (237–242) in the C-terminus of MARCH8 that are highly conserved between MARCH1 and MARCH8 are required for downregulation of TfR. siRNA knockdown of MARCH8 in HepG2 cells (a liver cell line) leads to the upregulation of TfR surface expression, indicating that endogenous levels of MARCH8 were sufficient to reduce TfR surface expression [96].

To date, studies of the MARCH proteins have focused on their function in the immune system. A recent study showed that MARCH8 is highly conserved between human, mouse, zebrafish, and* Xenopus*. Further examination of the function of MARCH8 in zebrafish and* Xenopus* showed that the expression MARCH8 is regulated during embryogenesis. Zebrafish MARCH8 expression is highly expressed in cleaved embryos; its expression decreases during gastrulation and then is upregulated during somitogenesis. Finally, MARCH8 expression appears to be restricted to the brain. Morpholino-oligonucleotide knockdown of MARCH8 in the developing embryo leads to abnormal development indicating that MARCH8 expression is vital to zebrafish embryonic development. Overexpression of wild type MARCH8 in the fertilized embryo leads to loss of cell adhesion, abnormal cell migration, and cell death, whereas overexpression of an inactive mutant of MARCH8 (W109A) did not have these effects, indicating that the level of active MARCH8 is extremely important during zebrafish embryogenesis. Overexpression of MARCH8 during* Xenopus laevis *embryogenesis leads to reduced adherence of the cells in the animal cap. These abnormalities in both zebrafish and* Xenopus* embryogenesis were due to the modulation of surface levels of E-cadherin by the E3 ubiquitin ligase activity of MARCH8 [103].

### 4.9. MARCH9

MARCH9 has a naturally occurring splice variant that does not contain a RING-CH domain [31]. Like many of the other MARCH proteins it has two transmembrane domains. Both the full length and the RING-less splice variant are expressed in most human tissues, with the full length being mostly highly expressed in the placenta, whereas the RING-less mutant is highly expressed in the lymph nodes, spleen, and lungs. In mice, MARCH9 mRNA is expressed in all tissues but appears to be highly expressed in the brain and the kidneys [67]. Other studies looking at the expression of MARCH9 indicate that it is predominantly expressed in B cells, T cells, and DCs [68]. The intracellular localization of MARCH9 is similar to MARCH4, that is, it is found in the Golgi but unlike MARCH4, it is mostly found in the* trans*-Golgi network [37]. Another study showed that MARCH9 localizes mainly to the lysosomes and only in cells expressing high levels was it localized to the* trans*-Golgi network [104].

*In vitro* ubiquitination studies to identify the E2 ubiquitin-conjugating enzyme used by MARCH9 found that the protein was unable to mediate ubiquitination with the following E2s: ubcH2, ubcH3, ubcH5a, ubcH6, ubcH7, Mm ubc6, or Mm ubc7. This indicates that some other E2 is coordinating with MARCH9 [37]. Overexpression of human full length MARCH9 results in reduced expressions of MHC I, HLA-DM, -DQ, -DR and -DO, CD4, ALCAM, and ICAM-1 at the cell surface, suggesting a possible role in immune regulation or signaling [6, 15, 31, 47]. The RING-less splice variant of MARCH9 was incompetent for downregulation of MHC I. Overexpression of full length MARCH9 leads to increased endocytosis of MHC I, sorting via MVBs (i.e., Vsp4 positive compartments) and subsequent trafficking and degradation of MHC I in lysosomes. Lysine residues in the C-terminus of MHC I HLA-2.1 are required for ubiquitination and downregulation of MHC I by MARCH9. Interestingly, knockdown of MARCH9 in HeLa cells did not lead to increased levels of MHC I heavy chains indicating that high levels of MARCH9 might be required for MHC I modulation, or that this might not be a true, physiological target [31].

ICAM-1 binds to the LFA-1 integrin and is involved in the formation of the immunological synapse during T cell activation and transendothelial migration of activated lymphocytes. Overexpression of full length MARCH9 leads to decreased surface levels of ICAM-1 due to ubiquitination of lysines present in the cytoplasmic tail of ICAM-1. MARCH9 causes ICAM1 to be degraded in lysosomes. Mutation of an aspartate residue to an asparagine residue found in the second transmembrane domain reduces the ability of MARCH9 to modulate MHC I but not ICAM-1 indicating that this region may be important for modulation of some targets. Similar to MHC I, RING-less MARCH9 is unable to downregulate the surface expression of ICAM-1 [104]. Interestingly, similar to K3, the DIRT domain is required for the function of MARCH9 [61].

MARCH9 is able to autoubiquitinate itself as well as dimerize with a RING-less splice variant of itself. Coexpression of full length MARCH9 and its RING-less splice variant leads to greater modulation of MHC I and ICAM-1, possibly by stabilizing the protein levels of full length MARCH9 [104]. This was surprising given that when KSHV K5 is coexpressed with a RING-less mutant, the RING-less mutant acts as a dominant negative [105]. Interestingly, murine MARCH9 is unable to downregulate ICAM-1 or MHC I K^b^, in C1498 cells (a mouse acute myeloid leukemia cell line), indicating that mouse and human MARCH9 may have cell type-dependent targets [67].

SILAC experiments and high throughput flow cytometry studies have been performed in B cells (Hs-Sultan cells, which are an Epstein Barr virus positive Burkitt lymphoma) overexpressing MARCH9 in order to identify additional targets. 44 different surface proteins were tested using the high throughput flow cytometry approach, which led to the identification of CD31, CD86, and CD166. These targets were downregulated to close the same efficiency as ICAM-1 by MARCH9. Three other proteins were downregulated to a greater extent compared to ICAM-1: Fc*γ*RIIB (an inhibitory receptor for the Fc portion of IgG antibodies that is involved in B cell homeostasis), a membrane bound IgD (mlgD; which is one to two isotypes of B cells antigen receptor expressed on mature B cells and is involved in B cells development and homeostasis), and CD155, also known as PVR as it was originally known as the receptor for Polio virus, which is also involved in establishing adherens junctions between epithelial cells, prevents NK killing of tumor cells and development of the humoral immune response. SILAC studies led to the identification 12 plasma membrane proteins that have reduced surface levels comparable or greater than ICAM-1 and had not already been identified by the flow cytometry screen. These hits are listed in Table 1.

Only Fc*γ*RIIB, SLAM, PTPRJ, ILT-2, and HLA-DQ were further validated as* bona fide* targets of MARCH9 by flow cytometry analysis. Pulse chase analysis of SLAM in HEK 293T cells in the presence and absence of MARCH9 indicates that MARCH9 specifically degrades the mature form of SLAM. This indicates that MARCH9 does not alter the maturation of SLAM but does cause the mature form of SLAM to be trafficked to MVBs and thus subsequently degraded by lysosomes [106].

Many studies have been performed to determine what amino acids or motifs are required in MHC II to enable MARCH9 to regulate some isotypes but not others. MARCH9 downregulates HLA-DQ but not HLA-DR or HLA-DP, when expressed at low levels but when expressed at extremely high levels it is able to target HLA-DP and -DR. HLA-DQ*β* contains the sequence ^215^LGLIIRQ^221^ in its C-terminus that is not found in HLA-DR*β*, but if this sequence is used to replace residues in the same location in HLA-DR*β*, mutant HLA-DR*β* is more efficiently targeted by MARCH9 than wild type HLA-DR*β* indicating that these residues play a significant role in recognition of targets by MARCH9 [97]. Overexpression of MARCH9 in HEK 293T cells also leads to the downregulation of HLA-DM. Similar to MARCH1 and 8, MARCH9 ubiquitinated HLA-DM*α* on K230 whether DM*α* is expressed on its own or as part of the DM*αβ* dimer in HEK 293T cells. MARCH9 is unable to downregulate surface levels of DM in Raji cells (a B cell line) but treatment of these cells with chloroquine leads to increased amounts of MARCH9 being present indicating that MARCH9 is being degraded in the lysosomes in these cells. DM*β* contains a functional tyrosine based endocytic motif. Modulation of DM in HEK 293T cells by MARCH9 requires both the DM*α* K230 and the DM*β* tyrosine based endocytic motif. The same sequences are required by MARCH1 to modulate HLA-DM but not MARCH8 [49]. HLA-DO is expressed mostly in B cells and has to be coexpressed with DM in order to form proper dimers; the requirement for DM can be overcome by mutating a proline residue in the groove of DO*α* to valine [107]. MARCH9 reduces the surface expression HLA-DO to a greater extent than MARCH1 or MARCH8. MARCH9 requires K225 in DO*β* for downregulation of DO*β* to a greater extent than MARCH1 and MARCH8 for DO downregulation, which is surprising as MARCH9 causes reduced ubiquitination of DO*β* compared to MARCH8. The cytoplasmic tail of DO*β* contains a di-leucine motif and a tyrosine based endocytic motif. Mutation of either or both the dileucine motif and the tyrosine motif in the presence of wild type DO*α* did not impact the ability of MARCH9 to down regulate mutant DO compared to wild type DO. Interestingly, mutation of both DO*α* K225 and the dileucine motif did reduce the ability of MARCH9 to down regulate mutant DO compared to when the DO*α* K225 is only mutated. This is not seen for MARCH1 or MARCH8. Overall, the ubiquitination of DO*α* is the most important factor in the downregulation of DO by any of the MARCH proteins studied. MARCH9 decreases surface expression HLA-DO but rather than degrading HLA-DO, it causes an intracellular accumulation of HLA-DO. This is different from the mechanism used by MARCH1 and MARCH8 to decrease surface expression of HLA-DO [47].

Given that Mult1, a ligand of the NK cell receptor NKG2D, is homologous to MHC I, it is not unsurprising that Mult1 is a target of MARCH9. Overexpression of human and murine MARCH9 leads to the internalization, but not degradation, of Mult1 in a manner that depends on the ubiquitination of lysines in the cytoplasmic tail of Mult1. Modulation of Mult1 by MARCH9 requires that MARCH9 interacts with Mult1 independent of the lysines in the cytoplasmic tail but requires part of the cytoplasmic tail to be present. Expression of mouse MARCH9 in C1498 cells (a murine acute myeloid leukemia cell line) prevents the cells from being lysed by activated NK cells to a greater extent than when mouse MARCH4 is expressed in C1498 cells [67].

### 4.10. MARCH10

To date only one study has been performed to better understand MARCH10. MARCH10, like MARCH7, is unusual for having no predicted transmembrane structures and an extended N-terminus. Similar to MARCH9, MARCH10 exists as two isoforms due to alternative splicing and are named 10a and 10b. MARCH10b does not contain a RING-CH domain but contains exons 1, 2, and 3 found in MARCH10a as well as another exon that is not found in MARCH10a that encodes a proline rich sequence [108]. Large-scale human transcriptome studies show that MARCH10 is highly expressed in the testes and most highly expressed in testicular germ cells [68]. While the expression of MARCH7 increases in developing rat spermatids, the expression of MARCH10 is restricting to elongating and elongated spermatids but is not expressed in the epididymal spermatozoa, that is, almost fully developed spermatids. MARCH10 expression is found in the cytoplasmic lobes, the annulus, and the principal piece, which is one of two segments found in the sperm tail, of elongating spermatids. When the isoforms were expressed in COS7 cells, MARCH10a formed a complex with microtubules (confirmed by treating the cells with nocodazole, a microtubule-depolymerizing agent), while MARCH10b was dispersed in the cytoplasm.* In vitro* ubiquitination studies showed that MARCH10a autoubiquitinates itself and it utilizes the E2 conjugating enzyme ube2b, which is expressed in spermatids. Autoubiquitination of MARCH10a ubiquitin ligase activity is dependent on the presence of intact microtubules. Ubiquitination of MARCH10a leads to its increased degradation by the proteasome. Overall this study indicates that MARCH10 is required for spermatid maturation [108].

### 4.11. MARCH11

Trying to better understand the E3 ubiquitin ligases involved in the ubiquitin-dependent protein-sorting pathway led to the discovery of MARCH11. MARCH11 is closely related to MARCH4 and MARCH9. It has two transmembrane domains, a RING-CH domain, an N-terminal proline rich domain, and a C-terminal tyrosine based endocytic motif, as well as a C-terminal PDZ domain. MARCH11 mRNA is predominately expressed in the testis and weakly in the brain and pituitary of rats. MARCH11 appears to be expressed during early rat spermatogenesis, that is, mostly in round spermatids. Within cells, MARCH11 is found in TGN and MVBs that are AP-1 positive and contain polyubiquitinated proteins and fucose glycoproteins, some of which are polyubiquitinated. The tyrosine-based motif in the C-terminus of MARCH11 is required for interaction with *μ*1-adaptin, whereas the PDZ domain of MARCH11 is required for interaction with Veli3, a PDZ protein that interacts with the PDZ domain of MARCH2 [32].

*In vitro* ubiquitination assays indicate that MARCH11 utilizes ubcH5b and ubcH5c as its E2 ubiquitin-conjugating enzymes. Given the similarity between MARCH11 and MARCH4, it is not surprising that MARCH11 can ubiquitinate CD4, a known target of MARCH4 [32]. Another target of MARCH11 is SAMT1, which is a member of a four transmembrane protein family that undergoes N-linked glycosylation. SAMT1 expression is restricted to developing spermatids, similar to MARCH11, and colocalizes to the TGN and MVBs in mouse spermatids. Overexpression of SAMT1-4 and MARCH11 in HEK 293T cells indicates that MARCH11 can interact with all four SAMT proteins and that each the SAMT proteins can oligomerize with themselves and each other. MARCH11 can ubiquitinate all four SAMT proteins but only significantly reduces the half-life of SAMT1. MARCH11 ubiquitinates the C-terminus of SAMT1, which is required for proper localization of SAMT1, leading to the degradation of mature SAMT1 via the lysosomes [109]. This evidence suggests that MARCH11 plays a role in ubiquitin-mediated protein sorting in TGN-MVB transport in developing spermatids.

## 5. Discussion

More than ten years after their identification, the physiological role of most MARCH proteins still requires further elucidation.  Table 2 summarizes the key features and experimentally validated targets for each of the MARCH proteins discussed in this review. There are many reasons why the study of the MARCH proteins continues to be a pressing challenge. The main difficulty is the tight regulation of most MARCH proteins, which causes them to be expressed at low concentrations and makes it difficult to study their endogenous function. Most studies on MARCH have therefore focused on utilizing overexpression systems, which carry the risk of introducing indirect effects. MARCH expression is primarily assessed via RT-PCR, which only indirectly measures protein expression via mRNA levels [12]. Additionally, there remains a pressing lack of* in vivo* models; transgenic mice models only exist for MARCH1, MARCH7, and MARCH8 [50, 90, 95]. It is possible that some of the proteins may be important in development and so cell type specific KOs would have to be generated to be able to study the functions of these proteins. Due to these technical difficulties, several aspects of the biology, and regulation of the MARCH proteins remain ill defined.

## 6. Transcriptional and Posttranslational Control

Transcriptional control of MARCH proteins is still not well understood. The best example of transcriptional control is the IL-10 mediated upregulation of MARCH1 [45]. Studying MARCH1 transcriptional control by IL-10 has yielded important insights into the physiological role of MARCH1 and the immunomodulatory potential of E3 ubiquitin ligases; hence, it could be useful model for studies of other MARCH proteins. Indeed, it seems that at least one other MARCH family member, MARCH2, may also be regulated at the transcriptional level in response to various cytokines (unpublished data).

Studies have shown that the MARCH proteins are expressed under specific conditions; for example, MARCH1 expression in DCs indicates that it is expressed at high levels in immature DCs and then downregulated in mature DCs [43, 53, 61]. MARCH4 is expressed only in certain tissues [31]. MARCH6 appears to be involved in sterol and thyroid hormone homeostasis and therefore is likely to undergo transcriptional regulation or posttranslational regulation [80, 81]. MARCH7 and MARCH10 are expressed during specific periods of spermatogenesis [88, 108]. Further studies need to be performed to identify the factors that regulate the transcriptional and/or posttranslational control of these proteins.

Many of the MARCH proteins appear to undergo auto-ubiquitination indicating that they regulate themselves [30, 38, 40, 41, 61, 104, 108]. Interestingly, CD83 and Tollip can inhibit MARCH1 activity indicating that there are proteins that can control the action of MARCH proteins [56, 57].

## 7. Targets of MARCH Proteins

Another issue that should be considered regarding MARCH proteins is the possibility that initial studies of MARCH function, which focused primarily on immunomodulatory function due to their presumed orthology to KSHV K3 and K5, suffered from a certain level of ascertainment bias [12]. New binding partners and functions continue to be identified for the MARCH proteins, and it seems that at least some MARCH proteins may very well have important functions outside of the context they were originally discovered in. For example, MARCH7 and 10 are involved in spermatogenesis [88, 108]. MARCH5 is involved in protecting cells from cytotoxicity due to the buildup of misfolded proteins or excess protein buildup [73–75].

The possibility of bias has made quantitative, proteomics-based methods an attractive option for identifying substrates of MARCH proteins. One such attempt using SILAC discovered that Bap31, a chaperone protein, predominately localized in the ER involved in forward transport and ERAD, associated with nearly all members of the MARCH family except for MARCH2 via their transmembrane domains. When MARCH8 was overexpressed in human foreskin fibroblasts (HFF) and HeLa cells, Bap31 was sequestered in the cell and prevented from going to the cell surface, providing more evidence that Bap31 is a substrate of MARCH proteins. This same approach failed to recognize ALCAM, a previously identified substrate of MARCH8, however, and identified CD9 as a substrate of MARCH8, a result that was not confirmed in additional experiments [37]. SILAC experiments have also been performed on cells overexpressing MARCH9 and many of the potential targets listed in Table 1 are still waiting to be validated [106]. Thus, substrates identified via SILAC should always be confirmed through more direct means.

## 8. Localization

Another important question that has required further elucidation is the localization of endogenous MARCH proteins in the cell. Sometimes, localization can have a direct effect on MARCH function, as it does in MARCH5-mediated regulation of TANK, which is dependent on MARCH5 localizing to the mitochondrial membrane [76]. Most information regarding subcellular localization of the MARCH proteins relies on overexpression of the MARCH protein, which may cause indirect effects. MARCH6, for example, is a particularly large protein with more than 900 amino acids, which localizes predominately in the ER when overexpressed. Although identification of its yeast homolog, Doa10, as an ER-associated protein supports this finding, it may also be that much of the MARCH6 being detected in the ER may be misfolded or is otherwise hindered from being transported out of the ER due to overexpression [41]. Approaches using antibodies that can bind to endogenous MARCH offer more direct evidence of endogenous MARCH localization in the cell.

## 9. MARCH Proteins and the Immune System

Like K3 and K5, the MARCH proteins were originally identified for their ability to downregulate a number of cell surface signaling molecules involved in the immune response [31]. Further studies have shown that only certain MARCH proteins play a significant immunomodulatory role. MARCH1 is expressed in immature dendritic cells and ultimately helps determine the development of T regs through its modulation of MHC II [55]. MARCH4 and MARCH9 regulate Mult1, a ligand for the NKG2D receptor expressed on NK cells preventing cells from being targeted for NK cell lysis [67]. MARCH5 modulates TANK leading to TLR7 signaling [76]. MARCH7 may play a role in immune tolerance [86, 90]. MARCH8 may have a role in the inflammatory response [99]. Further studies like the one performed for MARCH1 need to be performed to fully elucidate the functions of these proteins [55]. This indicates that MARCH proteins play a critical role in the immune response. Studies need to be performed to examine how the expression of the MARCH proteins changes upon infection with either viruses or bacteria to determine at which point these proteins are important. Once mouse models have been developed for the MARCH proteins, it will allow us to understand the true role of the MARCH proteins in the immune response.

## 10. MARCH5 and the Mitochondria

Published and unpublished work indicate that K5 has some effects on the mitochondria, such as leading to changes in mitochondrial structure and function (R. Karki, M. Renn, A. Ackermann, R. E. Means, unpublished data) [21]. In these studies, in addition to altering mitochondrial functionality with regards to respiration, K5 expression also caused mitochondrial fusion and protein accumulation. Other data demonstrate that this alteration might be having an impact on cell susceptibility to proapoptotic agents (F. Barriga, M. Renn, A. Ackermann, R. E. Means, unpublished data.) The mechanisms of this alteration are currently under exploration but are likely to parallel those of MARCH5.

To date work on MARCH5 indicates that it regulates the morphology of the mitochondria through its regulation of MFN1, MFN2, Drp1, and hFis1 [38, 39, 69–71]. MARCH5 plays a significant role in the bridging of the mitochondria and the ER as well as protecting cells from the buildup of cytotoxic proteins such as mSOD1, ΔNAT-3Q71, and S-nitrosylated LC1 [74, 75]. Finally, MARCH5 appears to be involved in the decision for neuronal cells to undergo stress-induced mitochondrial fragmentation leading to cell death [78]. These studies indicate that MARCH5 is a critical protein in deciding the fate of mitochondria in cells and so further work needs to be performed to establish how else MARCH5 regulates the mitochondria. Perhaps these studies on MARCH5 will shed new light on the function of some of the viral MARCH proteins such as K5.

## 11. MARCH Proteins in Other Metazoans

Very little is known about the expression of MARCH proteins in other metazoans. A gene duplication of human MARCH5 has been found in the* Oncorhynchus mykiss *(rainbow trout),* Coregonus maraena* (whitefish), and zebrafish genomes. Both genes span over 6 exons. MARCH5A appears to be a fish specific gene. MARCH5B has a splice variant (which excludes exon 5) that is expressed in specific tissues. Both genes have four transmembrane domains. MARCH5A plays a greater role in immune defense against viral hemorrhagic septicaemia virus (VHSV) compared to MARCH5B. Through computational analysis of several fish genomes, Rebl et al. also found that the zebrafish* D. rerio*, the three-spined stickleback* G. aculeatus*, the Japanese rice fish* Oryzias latipes*, the Japanese pufferfish* Takifugu rubripes*, and the spotted green pufferfish* Tetraodon nigroviridis* appear to encode the following eight MARCH genes: MARCH2, 4, 5, 6, 7, 8, 9, and 11. Zebrafish also encodes MARCH1. None of these have been experimentally validated. MARCH3 expression in the rainbow trout has been experimentally validated [110].

Our current knowledge of MARCH6 has been informed to a great degree by the work done on its yeast homolog, Doa10. The broad conservation between Doa10 and MARCH6 suggests that the two proteins may share similarities in structure, physiological role, and potential substrates. An approach used to analyze the number of transmembrane domains found in Doa10 has been found to be applicable to MARCH6 as well, providing a clear example of how studying MARCH proteins in other organisms could shed insight into their function in the humans [40]. Mouse models also hold significant promise for elucidating insight into MARCH function in humans, with many MARCH proteins sharing greater than 90% sequence identity in the conserved RING-CH domains or even the complete cDNA transcript between the two species.

As mentioned earlier, MARCH8 is expressed in zebrafish and* Xenopus laevis*. It has a unique role in embryogenesis through its modulation of E-cadherin [103]. This is the first evidence of a MARCH protein having a role outside of the immune system. Due to the unexpected role of MARCH8 in embryogenesis, it is likely that many of the other MARCH proteins may have similar roles and thus the generation of mouse models to study these proteins individually may prove to be more challenging than anticipated.

## 12. Conclusion

Since their discovery roughly ten years ago, MARCH proteins have been identified as important physiological regulators of various cellular processes, ranging from antigen presentation in maturing DCs to fission and fusion in the mitochondrial membrane. The current research shows us that MARCH proteins can bind to a wide variety of substrates and binding partners, including other membrane-bound proteins, SNAREs, and even structural elements of the cell like microtubules. Despite this evidence for many MARCH proteins having a physiological importance in the cell, they remain critically understudied. Future work on the MARCH proteins should focus on the binding partners, substrates, localization, transcriptional control, posttranslational modifications, and control of endogenous MARCH proteins. These topics have been difficult to grapple with conclusively due to the fact that most work on MARCH proteins have used overexpression systems due to their low expression levels in the native cell. As molecular tools, like antibodies, and genetic tools, like transgenic mouse models, continue to be developed, there is hope that these areas will become clearer in the future.

## Figures and Tables

**Table 1:** List of potential targets of MARCH9 identified in a SILAC experiment.

Potential target of MARCH9	Known function
PTPRA	Receptor tyrosine phosphatase
PTPRJ (CD148)	Receptor tyrosine phosphatase
PTPRF	Receptor tyrosine phosphatase
HLA-DQA	Present of processed antigens to T cells
HLA-DQB	Present of processed antigens to T cells
SLAM (CD150)	A self-ligand costimulatory receptor Receptor that recognizes Gram-negative bacteria Regulates the macrophage-killing machinery in phagosomes
ILT-2 (CD85j/LIR-1/LILRB1)	NK cell inhibitory receptor
FCRL2	Fc receptor related molecule
Plexin C1 (CD232)	Inhibitor of integrin-mediated adhesion of DCs
VAMP8	SNARE involved in endocytic vesicle fusion
TMEM2	Involved in myocardial and endocardial morphogenesis
TRAIL-R1	Immune surveillance

**Table 2:** Summary of the key features of each MARCH protein and its experimentally validated targets.

Protein	Number of TMs	Other structural features	Function	Experimentally validated targets	Tissues/cells/compartments it is highly expressed in	Regulated by	Key references
MARCH1	2	Tyrosine based intracellular sorting motif; tyrosine based endosomal sorting motif (YXXΦ); di-leucine based endosomal sorting motif C-terminal PDZ-binding domain	Regulator of antigen presentation; regulator of lymphocyte development	CD95 (Fas), TfR, HLA-DR, -DM, -DO; B7.2, CD98	Immature DCs, B cells, monocytes	IL-10, CD83, Tollip	[6, 43, 44, 46–50, 55–57]

MARCH2	2	Tyrosine based motif (YXXΦ); C-terminal PDZ-binding domain	Regulator of cell polarity; regulator of GCPRs signaling; modulator of intracellular trafficking of proteins	TfR, B7.2, BLG1, *β*_2_-adrenergic receptor, SX6, CAL	Heart	—	[31, 36, 63, 64, 66]

MARCH3	2	Tyrosine based motif (YXXΦ); C-terminal PDZ-binding domain	Modulator of intracellular trafficking of proteins	Bap31, SX6	Lung, colon, spleen	—	[31, 35, 37, 65]

MARCH4	2	Tyrosine based motif (YXXΦ); C-terminal PDZ-binding domain	Modulator of NK cell targeting; regulator of the development of the immune system	Bap31, MHC I, HLA-2.1, CD4, ALCAM, Mult1, SX4, CD81, B7.2	Brain, placenta, lungs, pancreas, some Golgi compartments	—	[15, 31, 37, 67]

MARCH5 (MITOL)	4	—	Regulator of mitochondrial morphology; regulator of neuronal cell death when under stress; positive regulator of TLR7 signaling; protects cells from cytotoxicity	MFN1, MFN2, Drp1, hFis1, TANK, mSOD1, ΔNAT-3Q71, S-nitrosylated LC1	Mitochondrial outer membrane, bronchial epithelial cells, thyroid, B cells	—	[38, 39, 70–77]

MARCH6 (TEB4)	14	TEB-Doa (TD) domain	Involved in recognition of specific targets for ERAD; associated with Cri-du-chat syndrome; regulator of response to cold temperatures; regulator of cholesterol production	D2, SM	ER membrane, nuclear envelope; most tissues	—	[40, 41, 80, 81]

MARCH7 (Axot)	0	N-terminus has serine/proline rich region; Tyrosine based motif (YXXΦ); RING-CH domain close to the C-terminus; non-classical importin-*α* nuclear localization signal (SKRPKL)	Confers stem cell properties; role in neuronal development; regulates immune tolerance	—	Mouse embryonic, neural and hematopoietic stem cells, human epithelial cells, human trophoblasts; rat testis; nucleus	—	[83, 85–88]

MARCH8 (c-MIR)	2	Tyrosine based motif (YXXΦ); C-terminal PDZ-binding domain	Regulator of the inflammatory response; regulator of CD4^+^ T cell development; regulator of apoptosis; possible restriction factor; Involved in zebrafish and *Xenopus laevis* embryogenesis	MHC I HLA-2.1, MHC II, CD95 (Fas), B7.2, TfR, CD166, CD44, CD88, CD98, IL1RAP, TRAIL-R1, SX4, CD166, Bap31, E-cadherin	Lung; developing zebrafish embryo; developing *Xenopus laevis* embryo; early endosomes, late endosomes, cell surface	—	[6, 15, 30, 31, 37, 46, 95, 96, 99–103]

MARCH9	2	C-terminal PDZ-binding domain; splice variant that does not contain a RING-CH domain	Regulator of the immune response; modulator of NK cell targeting	MHC I, HLA-DM, -DQ, -DR, -DO, CD4, ALCAM, ICAM-1, Fc*γ*RIIB, SLAM, PTPRJ, ILT-2, Mult1	Human placenta; mouse brain; mouse kidneys; B cells, T cells, DCs; *trans*-Golgi network	—	[31, 37, 67, 104, 106]

MARCH10	0	RING-CH domain close to the C-terminus; Exists as two isoforms due to alternative splicing	Regulator of spermatogenesis	—	Human testicular germ cells; developing rat spermatids	—	[68, 108]

MARCH11	2	Tyrosine based motif (YXXΦ); RING-CH domain close to the C-terminus; N-terminal proline rich region	Involved in rat spermatogenesis; involved in the ubiquitin-dependent protein-sorting pathway	CD4, SAMT1	Rat testis; TGN, MVBs	—	[32, 109]

TMs: transmembranes; TfR: transferrin receptor; DCs: dendritic cells; GCPRs: G-coupled proteins receptors; SX6: syntaxin 6; CAL: CFTF-associated ligand; ERAD: ER-associated degradation; D2: thyroid hormone activating type 2 iodothyronine deiodinase; SM: mammalian homolog of *Saccharomyces cerevisiae *Erg1; IL1RAP: IL-1 receptor accessory protein; TRAIL-R1: TNF-related apoptosis inducing ligand receptor 1; TGN: trans-Golgi network; MVBs: multivesicular bodies.
